# A multicenter, non-randomized, phase II study of docetaxel and carboplatin administered every 3 weeks as second line chemotherapy in patients with first relapse of platinum sensitive epithelial ovarian, peritoneal or fallopian tube cancer

**DOI:** 10.1186/1471-2407-14-937

**Published:** 2014-12-11

**Authors:** Yun Wang, Jørn Herrstedt, Hanne Havsteen, Rene DePoint Christensen, Mansoor Raza Mirza, Bente Lund, Johanna Maenpaa, Gunnar Kristensen

**Affiliations:** Department of Gynecologic Oncology, Norwegian Radium Hospital, Oslo University Hospital, Nydalen, PB 4953, 0424 Oslo, Norway; Department of Oncology, Odense University Hospital, Odense, Denmark; Department of Oncology, Herlev University Hospital, Herlev, Denmark; Research Unit of General Practice, Institute of Public Health, University of Southern Denmark, Odense, Denmark; Department of Oncology, Ålborg University Hospital, Ålborg, Denmark; Department of Obstetrics and Gynecology, School of Medicine, University and University Hospital of Tampere, Tampere, Finland; Department of Gynecologic Oncology and Institute for Medical Informatics, Norwegian Radium Hospital, Oslo University Hospital, Oslo, Norway

**Keywords:** Phase II study, Recurrent platinum-sensitive ovarian cancer, Docetaxel, Carboplatin, Toxicity

## Abstract

**Background:**

In patients with ovarian cancer relapsing at least 6 months after end of primary treatment, the addition of paclitaxel to platinum treatment has been shown to improve survival but at the cost of significant neuropathy. In the first line setting, the carboplatin-docetaxel combination was as effective as the combination of carboplatin and paclitaxel but with less neurotoxicity. This study was initiated to evaluate the feasibility of carboplatin with docetaxel as second line treatment in patients with ovarian, peritoneal or fallopian tube cancer.

**Methods:**

Patients with stage IC-IV epithelial ovarian, peritoneal or fallopian tube cancer were enrolled at the first relapse after at least 6 months since completion of the first line treatment. Docetaxel 75 mg/m^2^ was given as an one hour IV infusion followed immediately by carboplatin (AUC = 5) given as a 30–60 min. IV infusion on day 1 and repeated every 3 weeks for 6–9 courses. Primary endpoint was toxicity; secondary endpoints were response rate and the time to progression.

**Results:**

A total of 74 patients were included. Of these, 50 patients received 6 or more cycles, 13 received 3–5 courses and 11 received less than 3 courses. A total of 398 cycles were given. Grade 3/4 neutropenia was seen in 80% (59 of 74) patients with an incidence of febrile neutropenia of 16%. Grade 2/3 sensory peripheral neuropathy occurred in 7% of patients, but no grade 4 sensory peripheral neuropathy was observed. Sixty patients were evaluable for response. The overall response rate was 70% with 28% complete responses in the response evaluable patient population. Median progression-free survival was 12.4 months (95% CI 10.4-14.4).

**Conclusions:**

The three-weekly regimen of docetaxel in combination with carboplatin was feasible and active as second-line treatment of platinum-sensitive ovarian, peritoneal and Fallopian tube cancer. The major toxicity was neutropenia, while the frequency of peripheral neuropathy was low.

## Background

Epithelial ovarian cancer, the second most common gynecological malignancy, is the fifth leading cause of cancer-related death in women in the United States [1]. Most patients present with disease in advanced stage. Surgery followed by chemotherapy with carboplatin and paclitaxel has become the standard treatment [2]. Although most patients achieve a complete response, the majority will suffer a relapse and eventually die from the disease. Relapses occurring ≥ 6 months after end of first line treatment are considered platinum sensitive and are advised to be treated with a platinum based regimen [3].

The ICON4/AGO-Ovar-2.2 study [4] demonstrated improved survival by adding paclitaxel to a platinum agent in patients with platinum sensitive recurrent ovarian cancer. However, this combination is associated with significant neurotoxicity [5]. Furthermore, many women will suffer from persistent neuropathy from the initial taxane containing regimen, making re-treatment with paclitaxel a difficult endeavor [6]. Studies have shown that patients report motor neuropathy as the most unpleasant adverse effect of treatment [7] and the development of neuropathy is a major factor impairing quality of life [8], which can lead to early treatment discontinuation.

The combination of carboplatin and docetaxel was found to be associated with similar survival as the combination of carboplatin and paclitaxel in first line treatment of ovarian cancer [9]. The combination of carboplatin and docetaxel caused considerably less neurotoxicity than carboplatin-paclitaxel with grade ≥ 2 neurosensory toxicity in 11% versus 30% and grade ≥ 2 neuromotor toxicity in 3% versus 7% of patients. The positive clinical experiences with carboplatin plus docetaxel provide a strong basis for continued investigation of platinum/docetaxel based chemotherapy in the management of advanced ovarian cancer.

Only a few studies have evaluated docetaxel in combination with carboplatin as second-line combination chemotherapy for ovarian cancer [10, 11]. A phase II trial of docetaxel and carboplatin in recurrent platinum-sensitive ovarian, peritoneal or Fallopian tube cancer with a platinum-free interval of at least 6 months [10] enrolled 25 patients. Docetaxel 75 mg/m^2^ followed by carboplatin (AUC5) on day 1 was given every 3 weeks for 6 courses. Among the 23 evaluable patients, the overall response rate was 72% with 16 (64%) complete and 2 (8%) partial responses. Sensory neuropathy grade 1/2 was observed in 10 patients (40%), no grade 3/4 sensory or motor neuropathy was observed. Neutropenia was the most frequent grade 3/4 hematologic toxicity occurring in 15 patients (60%), but no episodes of febrile neutropenia was observed in this trial.

In order to evaluate the combination of carboplatin and docetaxel as treatment of platinum-sensitive recurrent ovarian, peritoneal and Fallopian tube cancers, the Nordic Society of Gynecologic Oncology (NSGO) performed a phase II trial in patients with a relapse ≥ 6 months after completion of first line treatment.

## Methods

### Study patients

Eligibility criteria included age ≥ 18 years, a histologically verified diagnosis of epithelial ovarian carcinoma, peritoneal or Fallopian tube cancer and disease progression 6 months or later after completion of first line treatment with carboplatin and paclitaxel. Measurable disease according to Response Evaluation Criteria in Solid Tumors (RECIST 1.0) or CA-125 assessable disease according to Gynecologic Cancer Inter Group (GCIG) criteria [12, 13]. Other key eligibility criteria included a WHO performance status of 0–2 and adequate bone marrow, renal and hepatic function. Patients with pre-existing peripheral neuropathy (National Cancer Institute Common Toxicity Criteria for Adverse Events [NCI-CTCAE version 2] > grade 1) were excluded.

The study was designed and carried out in accordance with good clinical practice, the declaration of Helsinki and national laws. The local ethics committee at each participating center approved the study (Danmark: Den Videnskabsetiske komite or Vejle og Fyns Amter; Norway: Regional Committees For Medical and Health Research Studies; Finland: Regional ethical review board). All patients gave their written informed consent before study entry.

### Study design

Patients were prospectively recruited into this single arm study. The main endpoint was toxicity, with special emphasis on the frequency of febrile neutropenia. Secondary endpoints were response rate and progression free survival. Treatment was given as a combination of docetaxel 75 mg/m^2^ intravenously followed by carboplatin (AUC = 5) based on the Calvert formula [14] using the glomerular filtration rate calculated according to the method of Cockroft and Gault [15]. Treatment was repeated every 3 weeks for 6–9 cycles unless progressive disease or unacceptable toxicity occurred. Written informed consent in compliance with the recommendations of the Declaration of Helsinki was obtained in all cases before inclusion. The study was registered with ClinicalTrials.gov (NCT02026921).

### Treatment plan and dose modification

All patients received premedication with corticosteroid and a serotonin receptor antagonist. With bone marrow recovery within 28 days, the patients were retreated with full dose of docetaxel. With recovery within 29–35 days, the dose of docetaxel was reduced to 60 mg/m^2^. When hematologic recovery was not achieved at day 35, the patient went off study. Only one dose reduction of docetaxel was allowed. In case of febrile neutropenia (ANC <1 × 10 ^9^ /L and fever ≥ 38.5°C) the dose of docetaxel was reduced to 60 mg/m^2^ and the dose of carboplatin to AUC4 in the subsequent cycles. Use of granulocyte colony stimulating factor (G-CSF) was not allowed on a routine basis, but could be used at the discretion of the investigator in case of myelotoxicity.

In case of peripheral neuropathy or oedema (grade 2) or gastrointestinal toxicity (diarrhoea, nausea, and vomiting grade 3) treatment was delayed until recovery for a maximum of 2 weeks and patients were retreated with docetaxel 60 mg/m^2^. In case of grade 3/4 peripheral neuropathy (motor or sensory), oedema grade 3/4 or any non-hematological toxicity grade 4, patients went off study.

### Clinical evaluation and assessments

Baseline assessments were performed within 14 days prior to study entry and included: hematological tests (full blood count and differential white cell count); biochemical profile (CA125, total serum bilirubin, alkaline phosphatases, AST or ALT and creatinine); physical examination (WHO performance status, weight, and height); radiological examination including chest X-Ray, abdominal-pelvic CT-scan. Other radiological examinations as indicated.

During chemotherapy, hematological tests were performed before each infusion and again on day 14 +/− 2 days. Biochemical tests and physical examination were performed before each infusion. Measurement of all lesions reported at baseline and screening for new lesions were performed every 9^th^ week with the same method as used at baseline. Evaluation of response was done according to RECIST 1.0 criteria. Adverse events were graded using NCI-CTCAE version 2.

Serious adverse events (SAEs) occurring within 30 days after chemotherapy were reported. Clinical follow-up was performed every 3 months for the first 2 years, every 6 months the following year and annually thereafter, respectively. Responses were verified at the first follow up visit when applicable. During follow-up, CA125 was measured at each visit until progression. All patients (including patients who were withdrawn from the protocol treatment) were followed according to this scheme.

### Statistical methods

Progression-free survival was defined as time from registration to progression or death by any cause. Survival curves were determined using Kaplan-Meier estimates. The sample size was chosen to allow for a relative narrow confidence interval for the frequency of febrile neutropenia.

## Results

### Patient characteristics and treatments

A total of 74 patients were enrolled into this phase II trial by 6 member institutions of NSGO from August 2004 to August 2005. Patient demographics are outlined in Table 1. The median age was 61 years (range, 27–79 years). The majority of patients had ovarian cancer (93%), FIGO stage III/IV disease (85%), and 60 patients (81%) had serous type histology. A total of 398 cycles were given. Eleven patients received only 1–2 courses, 13 received 3–5 courses and 50 received at least 6 courses. The reason for withdrawal before completion of the planned 6 cycles were progression in 4 patients, allergic reaction to carboplatin in 10, allergic reaction to docetaxel in 1, febrile neutropenia in 2, impaired performance status in 1, increased liver enzymes in 1, other toxicities in 4 and withdrawal of consent in 1 patient. Of patients who received only 1–2 courses, 1 stopped due to early progression, 2 due to toxicity, 1 due to allergic reaction to docetaxel and 7 due to allergic reaction to carboplatin.

**Table 1:** **Demographics and tumor characteristics**

Characteristics (n = 74)	No. of patients (%)
Age, median years (range)	61 (21-79)
Primary site	
Ovary	69 (93)
Peritoneal	2 (3)
Fallopian tube	3 (4)
Original stage	
Ic	5 (7)
IIb	1 (1)
IIc	5 (7)
IIIa	4 (5)
IIIb	5 (7)
IIIc	45 (61)
IV	8 (11)
Unknown	1 (1)
Tumor grade	
Well differentiate	11 (15)
Moderate well differentiate	21 (28)
Poorly differentiate/undifferentiate	36 (49)
Unknown	6 (8)
Histologic type	
Serous	60 (81)
Mucinous	2 (3)
Clear cell	2 (3)
Endometroid	5 (7)
Undifferentiate	1 (1)
Other	4 (5)
Response to firs line therapy	
Complete response (CR)	38 (51)
Partial response (PR)	8 (11)
Stable disease (SD)	2 (3)
Non-evaluated disease (NED)	26 (35)
Time from end of first line chemotherapy to relapse	
Median months (range)	15.7 (6-80.9)
Relapse between 6 to 12 months	26 (35.1%)
Relapse >12 months	48 (64.9%)

Dose reduction of docetaxel was done in 26 of 398 cycles (7%, 14 patients) and dose reduction of carboplatin was done in 5 of 398 cycles (1%, 4 patients). In total, dose reduction was done in 16 of 74 patients (22%).

Cycle prolongation of up to one week occurred in 20 patients due to the following reasons: neutropenia 5 patients, neutropenia and thrombocytopenia 1 patient, reduced performance status 3 patients, increased liver enzymes 1 patient, oedema 1 patient, intercurrent disease 4 patients and logistic reasons 5 patients. One patient had a cycle prolongation of more than one week due to an intercurrent disease.

### Toxicity

Non-hematologic toxicities of docetaxel-carboplatin are summarized in Table 2. Significant non-hematologic toxicities were uncommon, and overall the combination was well tolerated. Treatment-related sensory peripheral neuropathy was low, with grade 2 in 4 patients and grade 3 in 1 patient. Three patients (4%) suffered grade 1/2 motor peripheral neuropathy and none had grade 3/4.

**Table 2:** **Non-hematologic toxicity**

	Grade of toxicity (NCI-CTVAE grade v2.0)			
	1	2	3	4
	n(%)	n(%)	n(%)	n(%)
Arthralgia	23(31)	2(3)	1(1)	0(0)
Myalgia	29(39)	4(5)	0(0)	0(0)
Nausea	36(49)	12(16)	4(5)	0(0)
Vomiting	9(12)	7(9)	4(5)	0(0)
Mucositis/Stomatitis	21(28)	13(18)	0(0)	0(0)
Neurohearing	3(4)	1(1)	0(0)	0(0)
Neuromotor	2(3)	1(1)	0(0)	0(0)
Neurosensory	34(46)	4(5)	1(1)	0(0)
Edema	13(18)	4(5)	0(0)	0(0)
Fatigue	24(32)	18(24)	0(0)	0(0)
Change of taste	13(18)	4(5)	0(0)	0(0)
Anorexia/weight loss	2(3)	1(1)	0(0)	0(0)
Diarhoe	6(8)	0(0)	0(0)	0(0)
Nail changes	6(8)	5(7)	0(0)	0(0)
Rash	2(3)	1(1)	0(0)	0(0)
Epiphora	1(1)	2(3)	0(0)	0(0)

NCI-CTCAE: National Cancer Institute Common Toxicity Criteria for Adverse Events.

Nausea, arthralgia/myalgia, mucositis/stomatitis and fatigue were the most common non-hematologic toxicities, but rarely severe. Grade 3 emesis was reported in 4 patients (5%) and grade 3 arthralgia in one patient (1%). Grade 2 fatigue was reported in 18 patients (24%). Edema was not a significant clinical problem. Increased fluid retention grade 2 that did not require diuretic therapy was reported by 4 patients (5%).

Hematologic toxicity is presented in Table 3. Neutropenia and leukopenia were most frequently reported. The incidence of grade 3 and 4 neutropenia was 27% (20/74) and 53% (39/74), respectively. Febrile neutropenia was reported in 12 patients (16%), in 5 of these in the first course and in 3 in the second or third course. In 6 patients (8%) G-CSF was used after an episode of febrile neutropenia. Anemia was quite common but mild. Grade 1 /2 anemia occurred in 64 patients (86%), no grade 3 /4 anemia was reported. Nine patients (12%) received blood transfusion and 1 patient (1%) received erythropoietin support. Thrombocytopenia was rare, with only 7 patients (9%) experiencing this toxicity of whom 2 (3%) had grade 4.

**Table 3:** **Hematologic toxicity**

	Grade of toxicity (NCI-CTVAE grade v2.0)			
	1	2	3	4
	n(%)	n(%)	n(%)	n(%)
Leucocytopenia	15(20)	27(36)	21(28)	3(4)
Neutropenia	3(4)	5(7)	20(27)	39(53)
Febrile neutropenia	0(0)	0(0)	12(16)	0(0)
Thrombocytopenia	4(5)	1(1)	0(0)	2(3)
Anemia	44(59)	20(27)	0(0)	0(0)

NCI-CTCAE: National Institute Common Toxicity Criteria for Adverse Events.

### Response and survival

**Figure 1:**
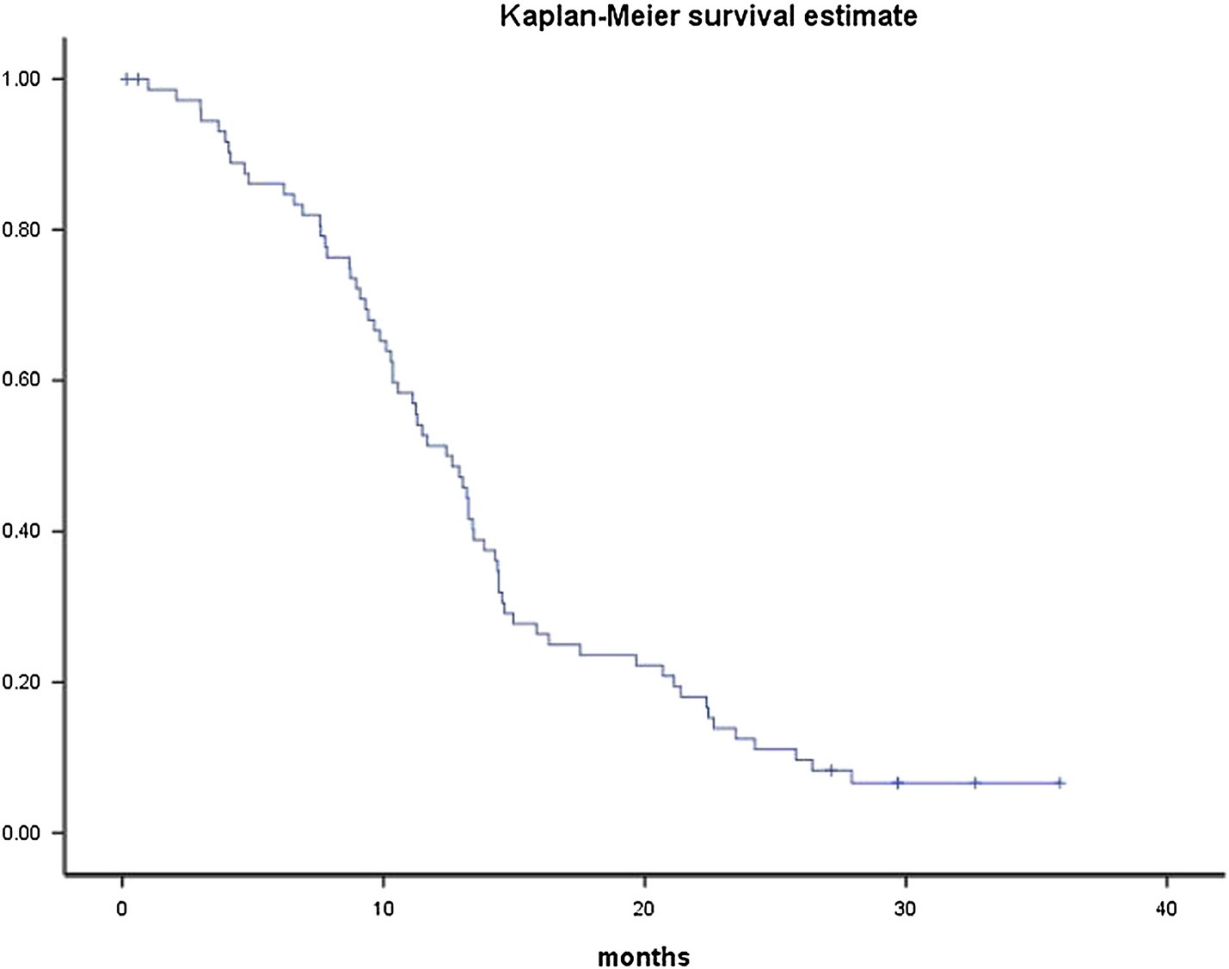
**Total progression-free survival in the intention-to-treat population (n = 74).**

Evaluation of response was performed in 61 patients with measurable disease. Seventeen achieved a complete response, 26 a partial response, 8 had stable disease and 10 had progression during treatment. The overall response rate was 70% (43/61) in the efficacy-evaluable patient population and 58% (43/74) in the intention to treat population. The median progression-free survival in the ITT population was 12.4 months (95% CI 10.4-14.4) as shown in Figure 1.

## Discussion

Overall the combination was well tolerated. Most cases of arthralgia and myalgia were mild. The most unpleasant side effects were mucositis/stomatitis grade 2 and fatigue grade 2, reported 18 and 24% of patients, respectively. The frequency of neurotoxicity was low. Neuropathy grade 2 and 3 was reported in only 6% and 1% of patients, respectively. This low frequency is in line with the findings in other studies on the combination of carboplatin and docetaxel in relapsed ovarian cancer [10, 16, 17]. In contrast, up to 27% moderate or severe neurological toxicity (≥ grade 2) has been observed in studies with the platinum-paclitaxel combination in recurrent ovarian, peritoneal and fallopian tube cancer [4, 18]. The study was restricted to patients with a maximum of grade 1 neurotoxicity at recruitment, thus we cannot evaluated how this combination would influence on more pronounced preexisting neuropathy. Unlike neuropathy induced by paclitaxel, which may manifest early during treatment, docetaxel-induced neutropathy generally does not appear until cumulative dose of docetaxel exceeds 600 mg/m^2^[17]. As docetaxel is associated with less neurotoxicity than paclitaxel and generally is delivered as a convenient 1-hour infusion, suitable for out-patient administration, it might be a good substitution for paclitaxel in order to decrease neuropathy in the treatment of recurrent ovarian cancer.

As anticipated, neutropenia was the major toxicity in the present study, occurring with grade 3/4 in 80% of all patients and was the main reason for dose reductions. Previous studies have demonstrated a high incidence of severe (grade 3/4) neutropenia when combining docetaxel with carboplatin in the treatment of ovarian and other gynecologic malignancies. A range of 33.3%-94% has been reported in first line [9, 19, 20], and of 60%-98% in second-line chemotherapy [10, 16].

Febrile neutropenia was reported in 11% of patients in the SCOTROC study [9] on first line chemotherapy. This did not compromise dose delivery or safety. In the present study, a higher frequency of febrile neutropenia (16%) was observed as could be expected in the second line setting. Febrile neutropenia usually occurred early in the treatment. The frequency of febrile neutropenia seen in our and another study [18] call for prophylactic use of G-CSF support when this combination is used in second line. Guidelines recommend prophylactic use of G-CSF in older patients (> 65 years-old) and in those with comorbidities if the risk of febrile neutropenia exceeds 10% [21]. In accordance with other studies [9, 10, 19], thrombocytopenia was infrequent and usually mild. We observed only two patients with thrombocytopenia grade 4. The low rate of thrombocytopenia indicates that docetaxel may have a thrombocyte sparing effect when combined with paclitaxel.

In first line treatment of ovarian cancer, the combination of carboplatin and docetaxel has been found as effective as the carboplatin-paclitaxel combination [9]. Although inter-study comparisons are problematic, the findings of an overall response rate of 70% in the response evaluable population and a median progression free survival of 12.4 months in the present study are in line with the findings in studies using carboplatin in combination with either paclitaxel or pegylated liposomal doxorubicin in second line treatment of ovarian cancer [4, 18, 22].

The relative high frequency of carboplatin hypersensitivity reactions is in accordance with previous reports [16, 18, 23] on repeated treatment with carboplatin in patients with relapsed ovarian cancer. It is of interest that this frequency seemed to be lower when these women were treated with the combination of carboplatin and pegylated doxorubicin compared to carboplatin and paclitaxel [18].

## Conclusions

The 3 weekly regimen of docetaxel in combination with carboplatin was feasible and active in second-line treatment for platinum-sensitive ovarian, peritoneal and fallopian tuber cancer with a low frequency of peripheral neuropathy. The major toxicity was neutropenia.
